# Adipogenesis Under Leptin Control: Mechanisms and Model-Specific Effects

**DOI:** 10.3390/ijms27114778

**Published:** 2026-05-26

**Authors:** Amna Abu Na’aim, Taty Anna Kamarudin, Nurul ‘Ain Arshad, Nurul Fariha Za’aba, Nur Aqilah Kamaruddin, Fairus Ahmad

**Affiliations:** Department of Anatomy, Faculty of Medicine, Universiti Kebangsaan Malaysia, Kuala Lumpur 56000, Malaysia; anamna6464@gmail.com (A.A.N.); tatykamarudin@ukm.edu.my (T.A.K.); nurulfariha98@gmail.com (N.F.Z.); nur.aqilah@ukm.edu.my (N.A.K.)

**Keywords:** leptin, adipogenesis, adipose tissue, leptin signaling, preadipocytes

## Abstract

Adipogenesis is one way by which adipose tissue expands in our body. It is a complex tightly regulated process involving differentiation of mesenchymal stem cells (MSCs) into mature, lipid-containing adipocytes. One of the byproducts of this mechanism is leptin, an adipokine that plays a pivotal role in regulating food intake and energy homeostasis. While the increase in leptin secretion in proportion to fat mass expansion shows that leptin functions as a downstream marker of adipogenesis, emerging studies suggest that leptin itself may influence the adipogenesis process and act as a regulator. However, despite much research done to explore this, its role remains incompletely understood and often contradictory, with studies reporting pro-adipogenic, anti-adipogenic, or neutral effects depending on experimental context. These discrepancies highlight the influence of factors such as leptin concentration, timing of exposure, cell type, adipose depot, and species differences. This review gathers current evidence on leptin’s role in adipogenesis, integrating findings from diverse experimental models and biological systems. We further examine the underlying molecular mechanisms and signaling pathways involved, aiming to clarify the context-dependent effects of leptin and identify key knowledge gaps to guide future research in adipose tissue biology and metabolic disease.

## 1. Introduction

Adipose tissue expands when lipid accumulation from excess calories exceeds its storage capacity [1]. The adipose tissue, mainly white adipose tissue, is distributed in different depots and can be classified into two main types: subcutaneous depots and visceral depots [2]. Subcutaneous depots include the gluteal, femoral, and subcutaneous abdominal regions, whereas visceral depots include the omental, mesenteric, and perirenal regions [3,4]. During excessive nutrient intake, fat accumulates in both visceral and subcutaneous depots, although the distribution and metabolic consequences differ [5].

This expansion occurs through two main mechanisms: hypertrophy, characterized by an increase in existing adipocyte’s size, and hyperplasia, which involves an increase in adipocyte number [6,7,8]. Hyperplasia arises through adipogenesis, a process that generates new adipocytes to accommodate excess lipid storage [9,10]. Adipogenesis is defined as the conversion of mesenchymal stem cells or precursor cells into mature, lipid-laden adipocytes and proceeds through two tightly regulated phases: commitment and differentiation [11,12]. This method of adipose tissue expansion is considered the “healthier” or preferred mechanism because it allows fat to be stored safely in subcutaneous tissue [13,14,15]. By distributing excess lipids into newly formed adipocytes, the body reduces lipid spillover into more harmful depots, such as visceral fat, peri-/epicardial fat, or deposited in non-adipose tissues, also known as ectopic fat [16,17,18].

During adipogenesis, leptin, an adipokine that plays a central role in regulating food intake and energy homeostasis is produced in proportion to fat mass [19]. Increasing evidence suggests that leptin is not merely a product or marker of adipogenesis, but also an active regulator of the adipogenic process itself [20,21,22]. This dual role highlights a complex feedback relationship in which leptin may modulate the very process from which it originates, thereby influencing adipose tissue expansion and remodeling. Notably, leptin’s effects on adipogenesis appear to be highly context-dependent, as the hormone may either promote or inhibit adipocyte formation depending on factors such as leptin concentration, sensitivity, depot-specific responses, metabolic stress, and inflammatory status.

This relationship is particularly relevant in obesity, where unhealthy white adipose tissue expansion is associated with impaired adipogenesis, while obesity progression is also characterized by leptin resistance, a condition in which the body becomes less responsive to elevated circulating leptin levels [23,24]. Together, these observations suggest a tightly interconnected relationship between leptin signaling and adipogenesis in adipose tissue dysfunction and metabolic disease. However, despite significant progress in understanding leptin biology, the precise role of leptin in regulating adipogenesis remains incompletely understood, with several key mechanistic aspects still underexplored.

Thus, this review aims to explore and examine the current evidence regarding the role of leptin in adipogenesis, integrating findings from different cell types, adipose depots, species, and experimental models, and to clarify the mechanistic pathways through which leptin regulates adipocyte proliferation, differentiation, and maturation. By addressing contradictory findings and mechanistic uncertainties, we seek to clarify the context-dependent actions of leptin in adipose tissue development and identify key knowledge gaps as directions for future research.

## 2. Adipogenesis and Leptin Production During Adipogenesis

### 2.1. Molecular Phases of Adipogenesis

Adipogenesis is a complex multi-step process that involves the differentiation of mesenchymal stem cells (MSCs) into mature, lipid-containing adipocytes. Two distinct phases have been recognized: commitment and terminal differentiation [25]. During the commitment phase, specific physicochemical signals restrict mesenchymal precursor cells to the adipocyte lineage, converting them into preadipocytes without notable morphological alterations [26]. This irreversible stage is followed by adipocyte differentiation, in which the committed preadipocytes undergo at least two rounds of cell division, also known as mitotic clonal expansion (MCE), before experiencing morphological changes that enable lipid accumulation [27,28].

#### 2.1.1. Commitment Phase

During the commitment phase, pluripotent stromal cells become restricted to the adipocyte lineage without exhibiting overt morphological changes [29]. Progenitor cells expressing markers such as alpha-smooth muscle actin (αSMA) and platelet-derived growth factor receptors α and β (PDGFRα/PDGFRβ) first commit to the preadipocyte lineage before undergoing terminal differentiation [30]. In this early stage, multiple factors and processes are crucial in regulating the transformation of adipocyte precursors, including cell cycle regulation, which is necessary for mitotic clonal expansion, cytoskeletal reorganization, and various factors influencing ribonucleic acid (RNA) metabolism [26].

Among the transcription factors identified during the early first wave are CCAAT/Enhancer-binding protein-β (C/EBPβ), CCAAT/Enhancer-binding protein-δ (C/EBPδ), Krüppel-like factor 4 (KLF4), Krüppel-like factor 5 (KLF5), cAMP response element-binding protein (CREB), sterol regulatory element-binding protein 1c (SREBP1c), and early growth response protein 2 (EGR2 or Krox20), which are induced rapidly by adipogenic stimuli [31,32,33,34]. It has been proposed that C/EBPβ and C/EBPδ jointly regulate the expression of both peroxisome proliferator-activated receptor-gamma (PPARγ) and CCAAT/Enhancer-binding protein-α (C/EBPα), the two key master regulators of adipogenesis in the second stage [35]. Dual phosphorylation by mitogen-activated protein kinase (MAPK) and glycogen synthase kinase 3β (GSK3β) promotes the phosphorylation and nuclear localization of C/EBPβ [36]. Activated C/EBPβ subsequently induces the transcriptional activation of PPARγ and C/EBPα, which are essential for DNA binding, mitotic clonal expansion, and terminal adipocyte differentiation [36].

In addition, the bone morphogenetic protein (BMP) signaling pathway plays a critical, instructive role in the commitment stage of adipogenesis. BMPs can trigger two main downstream signaling pathways, the canonical SMADs (SMAD1/5/8) and non-canonical MAPK pathways, and BMP4 is considered the primary driver of white adipocyte commitment [37,38]. Further studies have shown that downstream SMAD signaling plays a predominant role in lineage determination, whereas the P38/MAPK pathway exerts only partial influence on commitment efficiency [39]. In addition, zinc finger protein 423 (ZFP423) is recognized as a crucial, early transcription factor that determines the adipogenic fate, acting in part by amplifying the BMP signaling pathway [40]. ZFP423 functions as a coactivator of SMAD proteins (specifically SMAD1 and SMAD4), which are the downstream effectors of the BMP canonical signaling pathway [41].

Meanwhile, the wingless-type MMTV integration site (Wnt) family plays a complicated role in adipogenesis. Although some studies report upregulation of Wnt pathway components in proliferating preadipocytes, suggesting a potential role in early-stage commitment, the overall view is that Wnt signaling inhibits adipogenesis [42]. This inhibitory effect is primarily mediated through suppression of key adipogenic transcription factors, including PPARγ and C/EBPα, thereby maintaining precursor cells in an undifferentiated state [43,44]. Furthermore, Wnt-inducible factors such as WISP2 can negatively regulate BMP signaling, further limiting adipocyte differentiation [42,45,46].

In the undifferentiated state, preadipocytes express high levels of preadipocyte factor-1 (Pref-1), CCAAT/enhancer-binding protein homologous protein (CHOP), Krüppel-like factor (KLF), GATA transcription factors, and Wnt signaling molecules, all of which markedly decrease following adipogenic induction [36]. Persistent expression of any of these factors suppresses the adipogenic program and maintains the cells in the preadipocyte state.

#### 2.1.2. Differentiation Phase

The differentiation phase involves the transformation of preadipocytes into mature adipocytes capable of lipid storage and adipokine secretion [29]. This phase is primarily governed by the transcription factors PPARγ and C/EBPα [11,47,48]. PPARγ expression is induced during the transition from preadipocytes to adipocytes and is indispensable for adipocyte maturation, while C/EBPα plays a central role in terminal differentiation [49]. Full adipogenic differentiation requires cooperative activation of PPARγ and C/EBPα, leading to the transcription of mature adipocyte markers such as the insulin receptor, adiponectin, and fatty acid-binding protein 4 (FABP4) [30,50].

Other than these two, activator protein-1 (AP-1), a transcription factor complex composed mainly of Fos (c-Fos, Fra-1, Fra-2) and Jun (c-Jun, JunB, JunD) proteins, plays a complex, context-dependent, and often rapid, transient role in the initiation of adipogenesis with some members (like c-Jun and c-Fos) associated with promoting the mitotic clonal expansion [51,52,53,54]. Immediately after the induction of differentiation, induced AP-1 factors drive the cell cycle progression of preadipocytes, allowing them to re-enter the cell cycle from G0 and undergo proliferation [55].

During early adipogenesis, cytokines such as growth hormones and interleukins also activate the Janus kinase 2/signal transducers and activators of the transcription (JAK2/STAT) pathway [56]. Activated STATs, especially STAT3 and STAT5, promote the expression of key adipogenic transcription factors [57,58]. One of the suggested mechanisms by which STAT 3 acts is by binding to the distal region of the C/EBPβ promoter during the early phase of adipogenesis [59].

Meanwhile, the MAPK pathway, comprising extracellular signal-regulated kinase (ERK), p38, and c-Jun N-terminal kinase (JNK), plays a crucial, dual-faceted role in regulating adipogenesis, balancing preadipocyte proliferation with differentiation [60]. Furthermore, ERK activation has been shown to be necessary for the initiation of mitotic clonal expansion and adipogenesis through its involvement in cell cycle progression [36,60,61]. MAPK/ERK signaling initiates adipogenesis by activating C/EBPβ through sequential phosphorylation events. Activated C/EBPβ then promotes cell cycle progression and mitotic clonal expansion, both of which are essential for successful adipocyte differentiation [36]. Furthermore, one major downstream target of ERK1/2 signaling is AP-1 that regulates genes associated with cell proliferation and differentiation. In addition, ERK1/2 signaling can intersect with the phosphoinositide 3 kinase (PI3K)/Akt/mammalian (or mechanistic) target of rapamycin (mTOR)/ ribosomal protein S6 pathway through activation of p90 ribosomal S6 kinase (RSK) [62].

The PI3K/Akt pathway plays an important role in regulating cell cycle progression and adipogenesis. During cell cycle progression, the pathway modulates the expression of cyclin D and p27^KIP1^, two important regulators of the G1 phase [36]. Cyclin D promotes the transition from the G1 phase to the S phase, whereas p27^KIP1^ acts as a cell cycle inhibitor [63,64]. Through Akt activation, the pathway promotes cell proliferation by increasing cyclin D activity and suppressing inhibitory signals.

Akt also negatively regulates GSK3β. Normally, GSK3β promotes cyclin D1 degradation and inhibits phosphorylation of retinoblastoma protein (Rb), which is necessary for G1 phase progression [36,65]. Therefore, Akt-mediated inhibition of GSK3β stabilizes cyclin D1 and enhances Rb phosphorylation, facilitating cell cycle progression and mitotic clonal expansion during adipogenesis [36,65,66].

In 3T3-L1 preadipocytes, activation of the PI3K/Akt pathway promotes adipocyte differentiation, while inhibition of this pathway suppresses adipogenesis. One important downstream mechanism involves forkhead box O1 (FoxO1), a transcription factor regulated by insulin-induced Akt activation [36,67]. Akt phosphorylates FoxO1, causing its inactivation and exclusion from the nucleus [67,68,69]. This inactivation is important because active FoxO1 suppresses adipogenesis and inhibits clonal expansion [36].

Another pathway involved is the mTOR pathway, a central integrator that promotes adipogenesis mainly by enabling PPARγ/C-EBPα activation and lipogenesis. A study on 3T3-L1 cells showed that mTOR kinase activity is crucial for differentiation, as treatment with rapamycin or expression of kinase-dead mTOR suppresses adipogenesis and disrupts the positive feedback loop between C/EBPα and PPARγ, as well as PPARγ transactivation [70,71]. Meanwhile, Yu et al. demonstrated that under stimulation with an adipogenesis-inducing hormone cocktail, the PI3K/Akt and mTOR pathways showed strong activation with a similar temporal pattern, suggesting that they are functionally connected during the differentiation process [68]. When cells were treated with LY294002, a specific PI3K inhibitor, activation of both PI3K/Akt and mTOR/70 kDa ribosomal protein S6 kinase (p70S6K) pathways were blocked, indicating that PI3K activity is required for the activation of both signaling pathways. In contrast, rapamycin, which specifically inhibits mTOR, blocked only the mTOR/p70S6K pathway without affecting PI3K/Akt activation, suggesting that mTOR/p70S6K functions downstream of PI3K/Akt in the signaling cascade [68].

### 2.2. Adipogenesis and Adipocyte Turnover in Adulthood

In adulthood, the total number of adipocytes remains relatively constant in both lean and obese individuals, even following substantial weight loss, suggesting that adipocyte number is largely established during childhood and adolescence [72]. Although earlier studies proposed that adipose tissue expansion in adulthood occurs exclusively through hypertrophy, recent rodent lineage-tracing studies demonstrate that preadipocytes can also differentiate into new adipocytes, thereby contributing to adipose tissue expansion via hyperplasia [8,73,74]. Population and tracer studies show that an adult human’s adipocyte number is relatively stable but turns over at roughly 10% per year, implying continuous birth and death of adipocytes [75,76,77].

Using carbon-14 (^14^C) incorporation into DNA as a retrospective birth marker, Spalding et al. also demonstrated that approximately 10% of adipocytes are renewed annually throughout adulthood [78]. The balance between hypertrophy and hyperplasia is shaped by depot, age, inflammatory and hormonal context, and specialized progenitor cells [76,79,80]. For example, obesity or overnutrition can initially favor hypertrophy but later triggers death-coupled regenerative adipogenesis [81,82]. In terms of aging, an increased expression of cellular senescence-associated genes has been observed in adipose stem cells derived from aged adipose tissue, and preadipocytes isolated from older individuals showed reduced proliferative capacity and impaired differentiation [3,83,84].

### 2.3. Leptin Expression and Production in Adipocytes

Leptin expression is closely linked to adipocyte differentiation and maturation and serves as a marker of terminal adipocyte differentiation [85,86]. At the transcriptional level, leptin gene (*LEP*) expression is controlled by multiple transcription factors and cis-regulatory elements. Wrann and Rosen identified FOS-Like Antigen 2 (*FOSL2*), a component of the AP-1 transcription factor complex, as a key regulator of *LEP* expression [87]. *LEP* expression increased in parallel with *FOSL2* during human adipocyte differentiation and across multiple adipose depots in mice [88].

Additionally, dexamethasone treatment of mature human adipocytes upregulated both *LEP* and *FOSL2* expressions [89,90]. However, *FOSL2* expression was unaffected by fasting, indicating that it regulates adipocyte-specific leptin expression during differentiation rather than acute nutritional responses [91]. Other transcription factors, including C/EBP, Specificity protein 1 (Sp1), and Nuclear Factor Y (NF-Y), bind conserved motifs within the *LEP* promoter and are essential for basal and tissue-specific expression [92,93,94,95]. In addition, early growth response protein 1 (EGR-1) mediates insulin-induced *LEP* upregulation, while hypoxia-inducible factor 1 (HIF-1) activates *LEP* transcription under hypoxic conditions [96,97].

Leptin production is largely restricted to mature adipocytes which are mainly from white adipose tissue [98,99,100]. Leptin secretion positively correlates with adipocyte size and lipid content, with larger adipocytes producing higher levels of leptin [101,102]. As preadipocytes undergo differentiation and acquire mature adipocyte characteristics, leptin expression increases in parallel with lipid accumulation and activation of adipocyte-specific transcriptional programs [103]. Consequently, mature adipocytes represent the primary source of circulating leptin [104].

Collectively, these findings highlight that adipogenesis is not only a structural process of adipocyte formation but also a critical determinant of leptin production and secretion. This tight coupling between adipocyte maturation and leptin production suggests that leptin may function both as a downstream marker of adipogenesis and as an active regulator within adipose tissue.

## 3. Leptin and Adipocyte Dysfunction in Obesity

Leptin supposedly exerts multiple effects either centrally through hypothalamic regulation by controlling the appetite and energy intake, or indirectly via several hypothalamic–pituitary–target organ neuroendocrine axes such as the hypothalamic–pituitary–gonadal axis and hypothalamic–pituitary–adrenal axis [105]. However, typical obese individuals show hyperleptinemia but poor response to endogenous or injected leptin, a state called leptin resistance, which helps maintain excess body weight rather than correct it [23]. This state is known as leptin resistance, a condition in which the brain or peripheral tissues become less responsive to leptin stimulation, thereby failing to produce its expected effects. This state leads to a compensatory increase in leptin secretion in an attempt to maintain energy homeostasis; however, the elevated circulating leptin levels further exacerbate leptin resistance, creating a vicious cycle [10]. One of the mechanisms that may lead to the development of this resistance is the impairment of intracellular leptin signaling [62,106]. It could be due to neurons expressing leptin receptor (LepR) exhibiting reduced sensitivity to circulating leptin levels, thereby lowering the efficiency of leptin–receptor binding. Or it could be that cells expressing LepR may have impaired downstream signaling capacity [107]. Research has also found that chronically high leptin can trigger negative regulators such as suppressor of cytokine signaling 3 (SOCS3) and protein tyrosine phosphatase 1B (PTP1B), which contribute to leptin resistance in peripheral tissues, including muscle and adipose tissue [62,107,108,109]. Following leptin binding to its receptor, SOCS3 binds to tyrosine 985 (Tyr985) of LepR to inhibit JAK2 activity, whereas PTP1B dephosphorylates JAK2 directly [62,110,111,112]. These feedback mechanisms suppress downstream STAT3 and PI3K/AKT signaling pathways, thereby attenuating leptin signaling and promoting leptin resistance.

Furthermore, in obesity, adipose tissue experiences substantial remodeling, involving not only increases in adipocyte size and number but also infiltration by immune cells, development of tissue hypoxia, accumulation of extracellular matrix components, and dysfunction at the organelle level, including the endoplasmic reticulum, mitochondria, and lipid droplets within adipocytes [113]. These conditions, known collectively as adipose tissue dysfunction, are marked by increased accumulation of abdominal, visceral, hepatic, and ectopic fat, and a maladaptive adipokine secretion profile [114]. The continued unhealthy expansion of white adipose tissue under caloric excess and metabolic stress during obesity is also characterized by reduced adipogenesis, reduced thermogenic capacity, and diminished insulin sensitivity, resulting in cardiometabolic diseases [24]. Reduced adipogenesis in obesity mainly affects subcutaneous fat, forcing existing adipocytes to enlarge (hypertrophy), become inflamed, and release excess lipids, which drives ectopic fat and insulin resistance [42]. Multiple mechanisms such as senescent precursors, inflammatory signals like TNF- α, disrupted BMP4/Wnt pathways, and dysregulated p53/p16, converge to block differentiation [16,115,116].

Understanding the role of leptin in adipogenesis has important implications for addressing leptin resistance and adipocyte dysfunction in obesity. Although leptin is traditionally recognized for its central effects on appetite regulation, growing evidence indicates that it also modulates adipose tissue development at the cellular level [7]. Elucidating the signaling pathways through which leptin influences adipocyte proliferation and differentiation may help identify mechanisms underlying impaired leptin responsiveness in obesity. Clarifying the context-dependent actions of leptin may provide important insights into why adipose tissue expansion becomes maladaptive in obesity and why leptin-based therapeutic strategies have shown limited success in leptin-resistant states. Importantly, adipose tissue expansion through adipogenesis is considered metabolically favorable compared to hypertrophy, as it allows for safer lipid storage and reduces ectopic fat deposition [15]. Therefore, targeting leptin-mediated regulation of adipogenesis may represent a strategy to promote healthy adipose tissue remodeling rather than simply reducing fat mass.

## 4. Leptin Role in Adipogenesis

### 4.1. Primary Adipose-Derived Precursor Cells (Depot- and Species-Specific Effects)

#### 4.1.1. Rat Subcutaneous and Inguinal Adipose-Derived Preadipocytes and Stromal Vascular Cells

A study by Wagoner et al., using primary adipose-derived precursor cells, demonstrates that leptin exerts concentration-dependent and context-specific effects on adipose tissue biology [117]. The researchers examined leptin actions on preadipocytes and stromal vascular (SV) cells isolated from rat inguinal fat pads. In vitro, leptin displayed a biphasic effect on cell proliferation, with low concentrations (50 ng/mL) stimulating preadipocyte growth, while higher concentrations (250–500 ng/mL) significantly inhibited proliferation in both preadipocytes and SV cells. Despite these marked effects on precursor cell numbers, leptin did not influence adipocyte differentiation, as evidenced by unchanged glycerol-3-phosphate dehydrogenase (GPDH) activity.

In contrast to Wagoner et al., Machinal-Quélin et al. demonstrated that leptin exerts both proliferative and pro-adipogenic effects on primary rat subcutaneous preadipocytes [118]. A 24 h exposure to leptin (10 nM) significantly increased DNA synthesis, as shown by enhanced [^3^H]thymidine incorporation and confirmed by direct cell counting. Beyond proliferation, leptin also promoted adipogenic differentiation. Leptin-treated cells accumulated more lipid droplets after 48 h and showed increased GPDH activity, a classical marker of late adipocyte differentiation.

These findings indicate that leptin can act directly on adipose precursors to promote both cell expansion and differentiation, highlighting the importance of cell source, developmental stage, and experimental conditions in determining leptin responsiveness.

Palhinha et al. also supported these findings through their research. In adipose-derived stem cells (ASCs) isolated from subcutaneous and retroperitoneal fat depots of mice, leptin synergized with insulin to accelerate differentiation and increase lipid accumulation, with particularly strong effects observed in retroperitoneal ASCs [119]. They also found that leptin treatment increased expression of adipogenic markers (Caveolin-1 (CAV-1), PPARγ, SREBP1c, perilipin 1 (PLIN1)) while inducing a proinflammatory profile characterized by elevated tumor necrosis factor-α (TNF-α) and interleukin-6 (IL-6) expression. These findings suggest that leptin promotes adipogenesis in ASCs while simultaneously shaping the inflammatory environment of developing adipose tissue.

Velickovic et al. extended these observations using brown adipose tissue (BAT) and inguinal white adipose tissue (iWAT) from leptin-deficient (*ob*/*ob*) and wild-type mice [22]. In *ob*/*ob* animals, both brown and white adipose depots exhibited enlarged adipocytes and a reduced proportion of multilocular cells, indicating impaired remodeling and lipid organization. At the cellular level, progenitor cells from *ob*/*ob* mice showed markedly reduced adipogenic capacity, with lower lipid accumulation and decreased expression of FABP4, PPARγ, and adiponectin.

#### 4.1.2. Porcine Neonatal Subcutaneous Adipose Tissue

Species-specific differences in leptin action are evident in studies using porcine adipose tissue by T.G. Ramsay et al. [120]. Ramsay investigated leptin effects on stromal vascular cells isolated from neonatal pig subcutaneous fat. Across a wide range of concentrations, leptin alone did not significantly affect adipocyte differentiation, as measured by GPDH and lipoprotein lipase (LPL) activities, nor did it interfere with differentiation induced by insulin, dexamethasone, or insulin-like growth factor I (IGF-I). However, high-dose leptin (1000 ng/mL) significantly increased preadipocyte and stromal cell proliferation.

These results suggest that, in porcine adipose tissue, leptin primarily contributes to expansion of the precursor pool rather than directly promoting adipocyte maturation, potentially influencing adipose tissue growth through increased recruitment of new adipocytes.

#### 4.1.3. Primary Bovine Intramuscular Preadipocytes

In addition to porcine models, Yu et al. investigated leptin signaling in primary bovine intramuscular preadipocytes, a cell population critical for intramuscular fat deposition [21]. Both leptin and leptin receptor were highly expressed in intramuscular adipose tissue, and their mRNA levels were positively correlated across different developmental stages of preadipocyte differentiation. Functional manipulation revealed that leptin overexpression suppressed both preadipocyte proliferation and differentiation, whereas leptin knockdown enhanced these processes, indicating a direct inhibitory role of leptin in intramuscular adipogenesis.

These findings highlight that leptin’s effects on adipose precursor biology are highly context-dependent, varying not only with differentiation stage but also with adipose depot origin and species, and further emphasize that leptin may restrain lipid accumulation in specific fat depots rather than uniformly promoting adipogenesis.

### 4.2. Bone Marrow-Derived Mesenchymal Stromal Cells

#### 4.2.1. Human Bone Marrow Stromal Cell Line (Hms2–12)

Studies by Thomas et al., using bone marrow-derived stromal cells, revealed a distinct role for leptin in regulating lineage maturation rather than precursor expansion [121]. In the human hMS2-12 cell line, leptin did not affect cell proliferation but markedly altered differentiation outcomes. Early during differentiation (day 3), leptin transiently increased LPL mRNA expression, suggesting an early metabolic response. However, by day nine, leptin significantly reduced adipsin and leptin mRNA expression and decreased lipid droplet formation by approximately 50%, indicating inhibition of adipocyte maturation.

Notably, leptin did not alter the expression of lineage-commitment transcription factors such as PPARγ2, suggesting that leptin does not influence the initial fate decision between adipogenic and osteogenic lineages, but instead modulates maturation of already committed adipocyte precursors.

#### 4.2.2. Adult Bone Marrow Skeletal Stem Cells

Genetic studies further support a role for leptin in regulating mesenchymal lineage allocation. Yue et al. conditionally deleted the leptin receptor in bone marrow skeletal stem cells using Prx1-Cre; Lepr^fl/fl^ mice [122]. Loss of leptin signaling reduced adipogenesis while enhancing osteogenesis, indicating that leptin acts directly on undifferentiated mesenchymal stromal cells to bias lineage commitment toward adipocytes rather than osteoblasts. These findings demonstrate that leptin does not act directly on mature osteoblasts but instead regulates fate decisions at the level of early mesenchymal progenitors.

### 4.3. Immortalized Adipocyte Cell Lines

#### 3T3-L1 Preadipocytes and Adipocytes

Immortalized 3T3-L1 cells have yielded divergent findings regarding leptin’s role in adipogenesis, reflecting differences in experimental design, dose, and differentiation stage.

Kim et al. reported from their studies that leptin did not affect preadipocyte proliferation or differentiation in 3T3-L1 cells [123]. Their studied cells underwent normal 3-isobutyl-1-methylxanthine (IBMX)-dexamethasone-insulin (MDI)-induced differentiation, forming lipid-filled adipocytes. However, in fully differentiated adipocytes, leptin exerted strong metabolic effects by increasing glycerol release and reducing intracellular triglyceride accumulation, even in the presence of IGF-I. These findings suggest that leptin primarily regulates lipid metabolism rather than adipogenesis in this model.

In contrast, Zwirska-Korczala et al. found that leptin significantly reduced 3T3-L1 preadipocyte proliferation by approximately 36%, as assessed by [^3^H]thymidine incorporation, indicating an antiproliferative effect [124]. Ambati et al. further reported that leptin reduced cell viability, increased lactate dehydrogenase (LDH) release, suppressed lipid accumulation, decreased GPDH activity, and interfered with differentiation signals in 3T3-L1 cells, suggesting that leptin directly inhibits adipocyte differentiation and may exert cytotoxic effects under certain conditions [125].

Conversely, Palhinha et al. reported pro-adipogenic effects of leptin in 3T3-L1 cells [119]. In their study, leptin accelerated lipid accumulation, upregulated PLIN1, and increased expression of key adipogenic and lipogenic factors including PPARγ, SREBP1c, and CAV-1, particularly during early differentiation. Leptin also acted synergistically with insulin, indicating that its adipogenic effects may depend on metabolic context and co-stimulatory signals.

Collectively, these studies demonstrate that leptin’s effects on adipogenesis are highly dependent on cell type, developmental stage, species, and metabolic context, ranging from regulation of precursor proliferation and lineage commitment to modulation of lipid metabolism in mature adipocytes (Table 1).

## 5. Mechanistic Insight

The diverse and some contradictory effects of leptin on adipogenesis reported across experimental studies suggest that leptin does not function as a universal promoter or inhibitor of adipocyte formation, but rather as a context-dependent regulator whose actions are shaped by multiple interacting mechanisms. To better understand the mechanistic basis underlying these divergent findings, the current evidence can be broadly organized into three interconnected mechanistic frameworks: (1) intracellular signaling networks underlying leptin-mediated adipogenesis, (2) intrinsic cellular determinants of leptin-mediated adipogenic responses, and (3) microenvironmental and systemic regulation of leptin responsiveness in adipogenesis. Together, these mechanisms provide an integrated framework to explain why leptin may exert markedly different adipogenic effects across tissues, species, and physiological contexts [62,126,127].

### 5.1. Mechanism 1: Intracellular Signaling Networks Underlying Leptin-Mediated Adipogenesis

A major mechanism by which leptin influences adipogenesis is through the activation of several intracellular signaling cascades downstream of the leptin receptor [119]. In general, six LepR isoforms have been identified, generated through alternative splicing of the *LEPR* gene, which belongs to the type I cytokine receptor family [10,107]. Upon leptin binding, LepR undergoes autophosphorylation, initiating intracellular signaling cascades that involve JAK and downstream pathways such as STAT, the insulin receptor substrate (IRS)/PI3K, MAPK/ERK pathway, and adenosine 5-monophosphate-activated protein kinase (AMPK) [62]. Leptin receptor activation integrates intracellular cascades into a tightly interconnected network, though this crosstalk is cell-type specific.

Machinal-Quélin et al. demonstrated that leptin signaling in rat subcutaneous preadipocytes is highly stage-specific [118]. In confluent preadipocytes, leptin rapidly induced signal transducer and activator of STAT3 phosphorylation, whereas this response was lost following differentiation, despite continued LepR expression. In contrast, leptin-induced activation of the MAPK (p42/p44) pathway was maintained in both confluent and differentiated cells, indicating that MAPK signaling remains functional throughout adipogenesis. Upon LepR binding, activated JAK2 phosphorylates Tyr985 on LepR, generating a docking site for Src homology phosphatase 2 (SHP2). SHP2 subsequently recruits growth factor receptor-bound protein 2 (Grb2), leading to activation of downstream kinases, including Raf and MEK, that act as signaling intermediaries between SHP2 and ERK1/2, crucial initiators of adipogenesis [62,107,128,129]. However, downstream signaling consequences differed by developmental stage. Leptin stimulated AP-1 DNA-binding activity only in early preadipocytes, even though MAPK phosphorylation persisted in mature cells. This indicates a signaling shift from a STAT3/AP-1-responsive program in precursor cells toward a more restricted MAPK-dominant response after differentiation.

Extending these findings, Palhinha et al. demonstrated that leptin activates multiple converging signaling pathways in preadipocytes, including JAK2/STAT3 and PI3K/AKT and mTOR signaling [119]. For JAK2/STAT3 pathway, leptin binding dimerizes LepR and activates JAK2, which phosphorylates three key tyrosines (Tyr985, Tyr1077, Tyr1138) on LepR. Tyr1138 recruits STAT3 and consequently, the phosphorylated STAT3 form dimers and enters the nucleus to act as transcription factors and regulates genes such as POMC and SOCS3 [107,128,129]. Meanwhile, JAK2-dependent activation of IRS leads to PI3K activation and accumulation of 3,4,5-triphosphate (PIP3). The formation of PIP3 will then activates protein kinase 1 (PDK1) and Akt, activating the PI3K/AKT pathway in adipogenesis [62,107,130].

Further evidence supporting pathway-specific signaling comes from studies in bone marrow mesenchymal stromal cells [122]. In Prx1-Cre;Lepr^fl/fl^ mice fed a high-fat diet, deletion of LepR markedly reduced STAT3 phosphorylation, whereas Akt, ribosomal protein S6, and ERK phosphorylation remained unchanged, indicating preferential activation of the JAK2/STAT3 pathway by leptin in this context. Gene expression analysis showed reduced expression of C/EBPα and SOCS3 in which both are STAT3-responsive adipogenic genes. On the other hand, JAK2 activation promoted adipogenesis while suppressing osteogenesis, identifying JAK2/STAT3 as a critical signaling axis mediating leptin-driven lineage allocation in mesenchymal progenitors. Nevertheless, because functional inhibition of PI3K/AKT or MAPK signaling was not performed, the exclusive role of JAK2/STAT3 cannot be definitively concluded.

Importantly, Palhinha et al. found that leptin also activates mTOR signaling. They demonstrated in the study that pharmacological inhibition of mTOR using rapamycin completely abolished leptin-induced lipid droplet formation, establishing mTOR activation as an essential mediator of leptin’s pro-adipogenic effect [119]. These findings place the mTOR pathway downstream of leptin receptor signaling as a key metabolic checkpoint linking leptin signaling to lipid accumulation and adipocyte maturation.

In bovine intramuscular preadipocytes, Yu et al. reported that leptin suppresses adipogenesis through activation of the CAMKK2/AMPK signaling pathway, resulting in reduced triglyceride accumulation, lipid droplet formation, and expression of key adipogenic proteins. Mechanistically, leptin upregulated calcium/calmodulin-dependent protein kinase kinase 2 (CAMKK2), an upstream activator of AMPK, thereby enhancing AMPK signaling. Activation of this pathway subsequently inhibited the expression of proliferation-associated genes and proteins, including cyclin-dependent kinase 1 (CDK1), proliferating cell nuclear antigen (PCNA), and cyclin B1 (CCNB) [21]. Leptin also suppressed the expression of SREBP1c, a major regulator of lipogenesis, together with its downstream lipogenic targets such as FABP4, fatty acid synthase (FASN), and acetyl-CoA carboxylase alpha (ACCα) [21,36]. Through coordinated inhibition of both proliferative and lipogenic pathways, leptin ultimately attenuated the proliferation and adipogenic differentiation of intramuscular preadipocytes [21,131].

This highlights that leptin can engage distinct signaling hierarchies depending on depot and tissue type, resulting in divergent phenotypic outcomes. Another example in a different type of cell is, in fetal hypothalamic progenitors, leptin drives proliferation and favors neuronal over astrocyte differentiation, with dependence on ERK and STAT3 signaling [132]. While in cortical neural stem cells, leptin promotes proliferation and neuronal differentiation through coordinated MAPK/ERK, JAK2/STAT3, and PI3K/AKT [133]. Based on this evidence, divergent adipogenic outcomes may partly arise from differential activation of downstream LepR signaling pathways. While activation of PI3K/AKT/mTOR and MAPK/ERK pathways is generally associated with adipogenic promotion, activation of CAMKK2/AMPK signaling suppresses lipid synthesis and adipocyte differentiation. The balance between these signaling networks likely varies according to cell type, differentiation stage, leptin concentration, and metabolic context.

### 5.2. Mechanism 2: Intrinsic Cellular Determinants of Leptin-Mediated Adipogenic Responses

Beyond intracellular pathway activation, leptin responsiveness during adipogenesis is strongly influenced by the intrinsic cellular identity of adipose precursor populations, including their developmental origin, differentiation stage, transcriptional profile, and metabolic specialization.

In rat subcutaneous preadipocytes, leptin stimulates proliferation and accelerates differentiation, increasing GPDH activity and lipid droplet formation [118]. Meanwhile, in mature 3T3-L1 adipocytes, leptin suppresses lipid accumulation, reduces GPDH activity, and promotes lipolysis, illustrating anti-lipogenic actions in fully differentiated cells [123]. These contradictory findings may indicate that leptin can affect adipocyte developmental stages differently. As we have discussed before, adipogenesis is not a single event. It occurs through multiple sequential stages. Leptin responsiveness changes during differentiation could be due to LepR signaling changes and intracellular pathways shifting as discussed in mechanism 1, and also due to differences in metabolic demands [118,119,123,134,135]. Thus, the same leptin signal can produce completely different outcomes depending on the differentiation stage. In addition, because adipogenesis is controlled by a tightly coordinated transcriptional cascade, alterations in the availability and activity of adipogenic transcription factors may substantially influence cellular responsiveness to leptin.

The most important adipogenic transcription factor that could be influenced by leptin is PPARγ, the master regulator of adipogenesis. Studies by Velickovic et al., Machinal-Quélin et al., and Palhinha et al. showed that leptin increases PPARγ expression [22,118,119]. Together with C/EBPα, PPARγ plays a critical role in regulating the expression of enzymes involved in triglyceride synthesis, including lipin-1 and acyl-CoA diacylglycerol acyltransferase 1 (DGAT1), thereby promoting lipid accumulation in adipocytes [36]. However, a study by Thomas et al., using a bone marrow stromal cell line, did not observe changes in PPARγ expression following leptin treatment [121]. This suggests that the ability of leptin to stimulate master adipogenic regulators may depend on cellular context. Machinal-Quélin et al. further demonstrated that leptin can modulate early transcriptional regulators by rapidly increasing c-fos mRNA expression and enhancing AP-1 DNA-binding activity [118,136]. This may partly explain how leptin promotes early precursor proliferation in certain adipose depots. Furthermore, the same study showed that leptin alters the timing of transcriptional activation, as evidenced by transient upregulation of early adipogenic markers such as LPL and PPARγ2 in rat subcutaneous preadipocytes after 24 h of exposure, whereas responsiveness diminished at later stages. Overall, these findings suggest that leptin may be able to regulate different levels of the adipogenic hierarchy, including terminal differentiation markers and immediate-early regulators, according to the contexts as well as timing and duration of exposure [118,121,125]. In addition, the contradictory findings reported across studies may also indicate that different leptin concentrations can differentially regulate the adipogenic transcriptional hierarchy [117,118,120].

Going deeper, intrinsic adipocyte heterogeneity across depots could be one of the contributors to the diverse transcriptional responses elicited by leptin that we have discussed. Recent single-cell transcriptomic analyses further support the concept by showing that human white adipose tissue contains multiple adipocyte subpopulations with distinct transcriptional and metabolic profiles, including subsets enriched for lipogenic, lipolytic, insulin-signaling, or thermogenic gene programs [137,138]. Importantly, these subpopulations display strong depot-specific distribution patterns, with certain adipocyte subtypes preferentially localized to subcutaneous adipose tissue and others predominantly found in visceral depots [137]. Because leptin signaling interacts closely with pathways regulating lipogenesis, lipolysis, insulin sensitivity, and thermogenesis, differences in adipocyte subtype composition may substantially influence leptin responsiveness across depots [137,138,139]. This variability in adipogenic potential among adipocyte subpopulations may be due to differences in lineage-specific gene expression programs, where populations enriched in adipogenic genes exhibit greater differentiation capacity [138]. Supporting this concept, Schwalie et al. identified functionally distinct adipose progenitor populations and reported that cells enriched for F3/Cd142 tissue factor displayed limited adipogenic differentiation potential and exerted anti-adipogenic effects despite sharing an overall progenitor composition with other adipose precursor populations [138,140].

Furthermore, the studies discussed in previous sections employed adipocyte precursors derived from diverse anatomical origins, including stromal vascular fractions, intramuscular adipose tissue, and bone marrow-derived mesenchymal progenitors. Consistent with this variability, recent transcriptomic analyses support the existence of distinct progenitor populations that give rise to functionally specialized adipocyte subtypes [138]. For example, single-cell transcriptomic profiling of bone marrow stromal cells from C57BL/6 mice identified cellular clusters diverging along pro-adipogenic and pro-osteo/chondrogenic lineage trajectories [138,141]. Meanwhile, progenitor populations within perivascular depots appear particularly heterogeneous and lean more towards vascular remodeling in addition to adipogenesis [142,143,144]. This suggests that adipose progenitors from different depots are specialized to maintain the physiological functions of their associated tissues and organs, which may partly explain depot-dependent differences in leptin responsiveness. Furthermore, limited concordance between murine and human adipocyte subpopulations, together with evidence for multiple developmental origins of white and thermogenic adipocytes even within the same adipose depot, may further contribute to species- and depot-specific discrepancies observed in leptin-adipogenesis studies [137,145].

While multiple adipocyte subpopulations with distinct transcriptional and metabolic identities provide an important mechanistic basis for leptin action, adipogenesis occurs within a highly complex tissue environment. The intrinsic heterogeneity of adipocyte subpopulations across depots suggests that leptin responsiveness is further shaped by depot-specific microenvironments and systemic endocrine interactions.

### 5.3. Mechanism 3: Microenvironmental and Systemic Regulation of Leptin Responsiveness in Adipogenesis

Leptin’s effects on adipogenesis may differ depending on whether signaling occurs locally within adipose tissue or indirectly through systemic endocrine mediators. Depot-specific microenvironments, inflammatory cytokines, and interactions with hormones such as insulin and IGF-I may further modify leptin responsiveness, contributing to the heterogeneous findings observed across experimental models.

Wagoner et al. carried out experiments and found that leptin shows direct concentration-dependent effects on adipose precursor cells in cell culture, but in whole organisms its influence on adipose tissue is largely indirect, mediated through systemic circulating inhibitors rather than local adipose signaling [117]. To assess whether leptin exerts indirect effects in vivo, in their experiments, the authors examined serum and adipose tissue-conditioned media from leptin-infused rats. Notably, serum from leptin-treated animals inhibited precursor cell proliferation, despite containing very low leptin concentrations, suggesting that systemic leptin exposure alters circulating factors that suppress proliferation independently of leptin itself.

Meanwhile, in this study, conditioned medium from adipose tissue of leptin-treated rats did not differ from phosphate-buffered saline (PBS)-treated rats, which demonstrates that leptin in vivo does not seem to alter adipose paracrine signals that regulate proliferation or differentiation. Importantly, they found that adipose tissue-conditioned medium consistently enhanced preadipocyte differentiation regardless of treatment, emphasizing the intrinsic pro-adipogenic nature of adipose-derived factors.

Mechanistically, the heterogeneous effects of leptin on adipogenesis can be influenced by the complex microenvironment within adipose tissue depots. Physiological disturbances including obesity and aging are associated with altered circulating leptin and adiponectin levels, suggesting that adipose tissue cellular composition and endocrine activity undergo dynamic remodeling in response to metabolic and environmental stressors [139]. Importantly, not all white adipose tissue depots function identically or respond similarly to environmental stimuli. Adipose tissue is composed not only of mature adipocytes, but also adipose stem and progenitor cells, endothelial and vascular smooth muscle cells, fibroblasts, neurons, Schwann cells, and diverse immune cell populations, including macrophages, T cells, and B cells, which collectively form specialized adipose precursor niches [138,146,147,148]. This complex niche where many cell types and extracellular cues interact to control adipogenesis contributes to the heterogeneous adipogenic responses to leptin observed across adipose depots. For instance, extracellular matrix (ECM) surrounding adipocytes functions not merely as structural support but also as an active regulator of adipocyte fate determination. ECM composition and stiffness, and integrin-mediated signaling critically influence precursor proliferation, migration, adipogenic differentiation, and thermogenic programming [149,150,151,152]. Adipose ECM is enriched with collagens, fibronectin, and laminin, where basement membrane-associated collagens generally support adipocyte differentiation, whereas excessive accumulation of fibrillar collagens contributes to fibrosis and adipose dysfunction [153,154,155]. For example, type I collagen suppresses adipogenic differentiation through Yes-associated protein (YAP) activation, reducing adipogenic transcription factor expression and lipid accumulation [156]. Likewise, collagen XVIII deficiency has been associated with reduced adiposity and altered lipid metabolism, further highlighting ECM-mediated regulation of adipogenesis [153]. Notably, subcutaneous and visceral depots exhibit distinct ECM signatures [157,158,159]. Subcutaneous ECM generally provides a more permissive adipogenic environment, whereas visceral ECM tends to be stiffer and less supportive of adipogenic marker expression [157,159]. These depot-specific ECM properties may therefore partly explain the divergent effects of leptin observed across adipose depots.

In addition, increasing attention has focused on the bidirectional crosstalk between adipocytes and immune cells, particularly macrophages, in obesity-associated adipose tissue remodeling. Leptin and inflammatory cytokines form tightly interconnected feedback networks in which inflammatory mediators rapidly alter leptin production, while leptin itself stimulates expression of cytokines such as TNF-α, IL-6, interleukin-1 (IL-1) family members, and interferon gamma (IFN-γ) through signaling pathways including JAK2/STAT3, nuclear factor kappa B (NF-κB), p38MAPK, and ERK1/2 [160,161,162,163]. Conversely, pro-inflammatory cytokines including TNF-α and IL-1β acutely stimulate leptin secretion but may suppress *LEP* gene expression during chronic exposure [164]. Importantly, many inflammatory cytokines, including TNF-α, IL-1β, IL-6, IL-15, IL-17, IL-18, and oncostatin M, impair adipogenesis, whereas others such as IL-7 and IL-34 may promote adipocyte differentiation [165,166,167]. Thus, the inflammatory tone within depot-specific adipose tissue may substantially influence leptin responsiveness and contribute to depot-dependent adipogenic outcomes.

Supporting this concept, Emont et al. provide detailed cellular atlases of human and mouse subcutaneous and visceral white fat at single-cell resolution across a range of body weight [137]. The study revealed marked heterogeneity among adipocyte, progenitor, vascular, and immune cell populations across subcutaneous and visceral adipose depots in both humans and mice. Their findings demonstrated substantial species- and diet-dependent differences in immune cell composition, particularly within macrophage populations. In murine adipose tissue, high-fat diet exposure induced a dramatic expansion of macrophages, suggesting that obesity-associated immune remodeling may fundamentally alter local adipose signaling networks and potentially modify leptin-mediated regulation of adipogenesis [137]. The systemic effects may also help explain the mechanism behind leptin resistance where the systemic hyperleptinemia does not necessarily reflect effective local leptin signaling.

**Figure 1:**
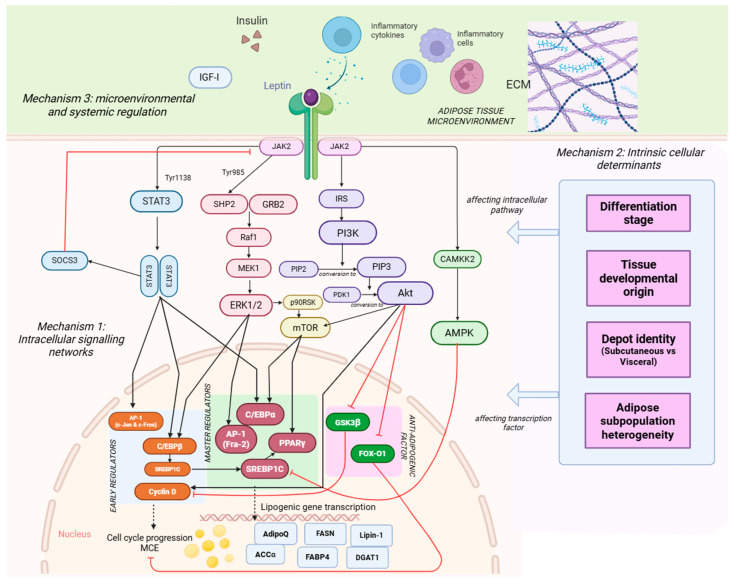
Schematic overview summarizing multiple interconnected leptin mechanisms regulating adipogenesis. The black arrows indicate activation processes while the red lines indicate inhibition. The dash arrow indicates the end adipogenic results. The area shaded orange within the cell reflecting mechanism 1 consists of four main pathways: the JAK2/STAT3 pathway, MAPK/ERK pathway, PI3K/Akt/mTOR pathway, and CAMKK2/AMPK pathway. The STAT3/SOCS3 pathway is the negative feedback inhibition pathway. The blue region inside the nucleus consists of early adipogenic regulators, while the green area includes master adipogenic regulators. The purple region inside the nucleus consists of anti-adipogenic transcription factors. The top is shaded green outside the cell reflecting mechanism 3 or adipose tissue microenvironment. Meanwhile, the box on the right, reflecting mechanism 2, consists of an intrinsic cellular identity that can affect the intracellular signaling and adipogenic transcription factors as depicted in the figure.

Altogether, current evidence indicates that leptin regulates adipogenesis through multiple interconnected mechanisms, which are summarized conceptually in Figure 1.

## 6. Leptin and Adipogenesis-Targeted Therapies in Obesity

Recent therapeutic strategies have focused on overcoming leptin resistance in obesity by targeting negative regulators of leptin signaling, such as SOCS3 and PTP1B. Pharmacological inhibitors of these molecules, including thiazolidinediones and trodusquemine, have been shown to suppress weight gain and reduce food intake in animal models, partly due to their ability to cross the blood–brain barrier (BBB) and restore central leptin responsiveness [168]. Given the limited efficacy of leptin monotherapy in obesity, largely due to resistance, increasing attention has been directed toward combination therapies of leptin and leptin sensitizers. Moreover, several agents have been reported to improve leptin sensitivity through complementary mechanisms. Peripheral histone deacetylase (HDAC) 6 inhibition using tubastatin, as well as treatment with metformin, has been shown to enhance central leptin sensitization [109,169]. Similarly, add-on therapies such as long-acting leptin analogues and exendin-4 contribute to the restoration of leptin responsiveness [169].

Natural compounds have also emerged as promising leptin sensitizers. For example, celastrol, a natural friedelane pentacyclic triterpenoid isolated from some celastraceae plants such as *Tripterygium wilfordii* has been identified as a potent anti-obesity agent that induces significant weight loss in diet-induced obese (DIO) mice [23,170]. Although its precise molecular mechanism remains unclear, celastrol appears to enhance leptin sensitivity, at least in part, through upregulation of interleukin-1 receptor 1 (IL1R1) expression in the hypothalamus [23]. Similarly, withaferin A has been shown to improve leptin responsiveness partly via sensitizing LepR signaling and increasing hypothalamic STAT3 phosphorylation in DIO mice [23,171].

Recently, increasing attention has been directed toward the anti-obesity potential of natural products that target adipogenesis and lipid accumulation. Many bioactive compounds have been reported to suppress adipocyte differentiation through multiple mechanisms, including induction of apoptosis, cell cycle arrest, or delayed cell cycle progression, and interference with adipogenic transcription factor cascades or intracellular signaling pathways during the early stages of adipogenesis [12,30,36]. One major therapeutic target is the AMPK pathway, a central regulator of cellular energy metabolism and adipogenesis. Several natural compounds act as indirect AMPK activators, including resveratrol, cryptotanshinone, medicarpin, L-theanine, crocin, sulforaphane, and platycodin D [30]. These compounds have been shown to promote brown and beige adipogenesis and/or suppress white adipogenesis in an AMPK-dependent manner [30,172]. Other phytochemicals exert anti-adipogenic effects by modulating signaling pathways involved in adipocyte differentiation. For instance, dehydroleucodine, caffeine, sulforaphane, and bisdemethoxycurcumin suppress Akt activation, whereas cocoa-derived compounds, caffeic acid phenethyl ester, piceatannol, and dieckol inhibit MAPK phosphorylation [12]. In addition, water extract of *Hibiscus sabdariffa* L. has been shown to inhibit ERK phosphorylation [12,173].

Natural compounds may also interfere with adipogenesis by regulating key adipogenic transcription factors. Several phytochemicals, including dehydroleucodine, apigenin, piceatannol, dieckol, and *Rehmannia glutinosa*, suppress C/EBPβ expression, an early regulator of adipocyte differentiation. Retinoic acid has been reported to inhibit C/EBPβ-induced PPARγ expression, thereby blocking downstream adipogenic programming [12,36]. Furthermore, compounds such as piperine, guggulsterone, and curcumin downregulate PPARγ expression, ultimately attenuating adipocyte maturation and lipid accumulation [12,36].

In addition, as discussed previously, adipogenesis may exert either beneficial or detrimental effects depending on the physiological context. Recent studies increasingly emphasize that metabolic health is influenced not merely by the quantity of adipose tissue, but also by the manner in which adipose tissue expands. This has led to growing interest in whether selective modulation of adipogenesis could dissociate obesity from its associated metabolic complications. For example, thiazolidinediones, which act as PPARγ agonists, are known to promote adipogenesis and induce adipose tissue “beiging” [174,175]. Experimental evidence suggests that adequate PPARγ activity and de novo adipogenesis from specific precursor populations are essential for healthy white WAT remodeling. In mice, stimulation of adipogenesis from PDGFRβ^+^ precursor cells improved visceral WAT function without increasing overall adiposity, whereas depletion of PPARγ in adipose progenitors impaired adipogenesis and resulted in pathological WAT expansion [75,174,175,176].

These findings highlight the dual nature of adipogenesis as a therapeutic target. Modulating adipogenesis is double-edged; promoting it in the right depot and lineage can improve metabolic health, but excessive or mislocalized adipogenesis promotes obesity and insulin resistance [174,177,178]. Therefore, stimulating adipogenesis should not be viewed simply as an anti-obesity strategy aimed at reducing body weight. Rather, selectively enhancing healthy, depot-specific adipogenesis, particularly when combined with thermogenic activation of brown or beige adipocytes, may help uncouple obesity from its metabolic complications [175]. Currently, blocking maladaptive white adipogenesis and supporting brown/beige adipogenesis remain the widely pursued anti-obesity strategies [12,30,36]. Current evidence is largely preclinical, and safely exploiting adipogenesis for therapy will require precise targeting of cell types, depots, and pathways.

## 7. Conclusions

In conclusion, current evidence demonstrates that leptin is not merely a passive marker of adipocyte maturation, but an active and highly context-dependent regulator of adipogenesis. Across a wide range of experimental models, leptin has been reported to promote, suppress, or exert minimal effects on adipocyte proliferation and differentiation, highlighting the complexity of leptin-mediated regulation within adipose tissue biology. Rather than representing conflicting observations alone, these divergent findings collectively suggest that leptin responsiveness is determined by the interaction of multiple mechanistic layers operating at intracellular, cellular, and tissue-environmental levels.

This review proposes that the heterogeneous effects of leptin on adipogenesis can be broadly understood through three interconnected mechanisms. First, leptin activates multiple intracellular signaling networks, including JAK2/STAT3, PI3K/AKT/mTOR, MAPK/ERK, and CAMKK2/AMPK pathways, whose relative dominance may differ according to cell type, differentiation stage, and metabolic state. Second, intrinsic cellular determinants such as developmental origin, adipogenic transcriptional hierarchy, lineage commitment, and adipocyte subtype heterogeneity strongly influence how precursor cells respond to leptin stimulation. Third, leptin responsiveness is further modulated by depot-specific microenvironments and systemic endocrine conditions, including extracellular matrix composition, inflammatory cytokines, immune–adipocyte interactions, and obesity-associated tissue remodeling. Together, these mechanisms provide an integrated framework explaining why leptin produces markedly different adipogenic outcomes across species, adipose depots, and experimental settings.

Importantly, understanding how leptin regulates adipogenesis has substantial physiological and clinical relevance. Impaired adipogenesis and leptin resistance are both central features of obesity-associated adipose tissue dysfunction, contributing to hypertrophic adipocyte expansion, ectopic lipid accumulation, chronic inflammation, and insulin resistance. Clarifying how leptin signaling influences adipose precursor fate and tissue remodeling may therefore improve understanding of why adipose tissue expansion becomes maladaptive during obesity and why leptin-based therapeutic strategies often fail in hyperleptinemic states.

## 8. Future Directions

The limitation across current studies is the large variability in leptin concentration, exposure duration, differentiation stage examined, adipocyte source, and species and depot origin. Notably, many in vitro studies employ leptin concentrations that exceed circulating levels reported in vivo, which may contribute to the divergent outcomes observed across models [179,180]. Future studies should prioritize standardized experimental frameworks incorporating physiologically relevant leptin concentrations, temporal exposure analyses, and clearly defined stages of adipocyte differentiation to improve comparability across models.

Next, advanced single-cell and spatial multi-omics approaches may help identify depot-specific leptin-responsive adipocyte subpopulations and clarify how signaling heterogeneity contributes to divergent adipogenic outcomes [181,182]. Future mechanistic studies should also investigate the dynamic crosstalk between LepR-activated signaling pathways rather than examining individual pathways independently. The interaction between leptin signaling and adipose tissue mechanotransduction pathways, including ECM stiffness and YAP activation, represents an emerging area requiring further investigation [183,184,185]. In addition, greater emphasis on human adipose tissue models and patient-derived precursor systems is needed to improve translational relevance and address species-dependent discrepancies [137].

Ultimately, integrating signaling biology, adipose tissue heterogeneity, and microenvironmental regulation into unified experimental frameworks will be critical for resolving the complex and context-dependent role of leptin in adipogenesis and for identifying novel therapeutic targets in obesity and metabolic disease. Future therapeutic strategies may benefit from targeting leptin responsiveness and adipose tissue remodeling pathways rather than leptin supplementation alone [12,30,186].

## Figures and Tables

**Table 1:** Experimental findings describing leptin regulation of adipogenesis across species, adipose depots, and cellular models.

Study	Species/Model	Cell Type or Adipose Depot	Leptin Exposure/Context	Effect on Adipogenesis	Mechanistic Notes
Wagoner et al., 2006 [117]	Rat	Primary preadipocytes and stromal vascular cells (inguinal fat)	Low leptin (50 ng/mL) vs. high leptin (250–500 ng/mL)	Biphasic proliferation: low dose ↑ proliferation; high dose ↓ proliferation; no change in differentiation	Dose-dependent effects suggest leptin regulates precursor pool size rather than differentiation
Machinal-Quélin et al., 2002 [118]	Rat	Primary subcutaneous preadipocytes	10 nM (≈160 ng/mL) leptin exposure (24 h)	↑ proliferation and ↑ adipogenic differentiation	MAPK/AP-1 activation; increased lipid accumulation and GPDH activity
Palhinha et al., 2019 [119]	Mouse	Adipose-derived stem cells (subcutaneous and retroperitoneal depots)	Leptin + insulin	↑ adipogenesis and lipid accumulation, stronger in retroperitoneal depot	↑ PPARγ, SREBP1c, CAV-1, PLIN1; pro-inflammatory cytokines (TNF-α, IL-6)
Velickovic et al., 2023 [22]	Mouse (*ob*/*ob* vs. wild type)	Brown adipose tissue and inguinal WAT progenitors	Genetic leptin deficiency	↓ adipogenic capacity and impaired adipose remodeling	↓ FABP4, PPARγ, Adiponectin expression
Ramsay, 2005 [120]	Pig	Stromal vascular cells (neonatal subcutaneous fat)	Wide leptin range (up to 1000 ng/mL)	No effect on differentiation; high leptin ↑ precursor proliferation	Suggests leptin expands precursor pool without promoting maturation
Yu et al., 2024 [21]	Bovine	Primary intramuscular preadipocytes	Leptin overexpression vs. knockdown	Leptin overexpression ↓ proliferation and ↓ differentiation; knockdown ↑ adipogenesis	Indicates depot-specific inhibitory role of leptin
Thomas et al., 1999 [121]	Human	Bone marrow stromal cell line (hMS2-12)	Early differentiation exposure	↓ adipocyte maturation and ↓ lipid accumulation	↓ adipsin expression; no change in PPARγ2
Yue et al., 2016 [122]	Mouse	Bone marrow skeletal stem cells	LepR deletion (Prx1-Cre;Lepr^fl/fl^)	↓ adipogenesis and ↑ osteogenesis	Leptin signaling biases mesenchymal lineage commitment to adipocytes
Kim et al., 2008 [123]	Mouse	3T3-L1 preadipocytes and adipocytes	Standard differentiation conditions	No effect on proliferation or differentiation	Leptin regulates lipid metabolism in mature adipocytes
Zwirska-Korczala et al., 2007 [124]	Mouse	3T3-L1 preadipocytes	Leptin treatment	↓ preadipocyte proliferation	Antiproliferative effect
Ambati et al., 2007 [125]	Mouse	3T3-L1 cells	Leptin exposure during differentiation	↓ adipogenesis and ↓ lipid accumulation	↓ GPDH activity; cytotoxic effects
Palhinha et al., 2019 [119]	Mouse	3T3-L1 cells	Leptin + insulin	↑ adipogenesis and lipid accumulation	↑ PPARγ, PLIN1, SREBP1c expression

↑, increased; ↓, decreased. Mitogen-activated protein kinase/activator protein-1 (MAPK/AP-1), glycerol-3-phosphate dehydrogenase (GPDH), peroxisome proliferator-activated receptor-gamma (PPARγ), sterol regulatory element-binding protein 1c (SREBP1c), caveolin-1 (CAV-1), perilipin 1 (PLIN1), tumor necrosis factor-α (TNF-α), interleukin-6 (IL-6), fatty acid-binding protein 4 (FABP4), adiponectin, leptin receptor (LepR).

## Data Availability

No new data were created or analyzed in this study. Data sharing is not applicable to this article.
